# Germline Testing in a Cohort of Patients at High Risk of Hereditary Cancer Predisposition Syndromes: First Two-Year Results from South Italy

**DOI:** 10.3390/genes13071286

**Published:** 2022-07-21

**Authors:** Francesco Paduano, Emma Colao, Fernanda Fabiani, Valentina Rocca, Francesca Dinatolo, Adele Dattola, Lucia D’Antona, Rosario Amato, Francesco Trapasso, Francesco Baudi, Nicola Perrotti, Rodolfo Iuliano

**Affiliations:** 1 Medical Genetics Unit, Mater Domini University Hospital, 88100 Catanzaro, Italy; colaoemma@unicz.it (E.C.); fernandafabiani@libero.it (F.F.); valentinarocca68@gmail.com (V.R.); francesca.dinatolo@studenti.unicz.it (F.D.); adele.dattola@studenti.unicz.it (A.D.); dantona@unicz.it (L.D.); rosario.amato@unicz.it (R.A.); trapasso@unicz.it (F.T.); baudi@unicz.it (F.B.); perrotti@unicz.it (N.P.); 2 Department of Health Sciences, Campus S. Venuta, University Magna Graecia of Catanzaro, 88100 Catanzaro, Italy; 3 Stem Cells and Medical Genetics Units, Tecnologica Research Institute and Marrelli Health, 88900 Crotone, Italy; 4 Department of Experimental and Clinical Medicine, Campus S. Venuta, University Magna Graecia of Catanzaro, Viale Europa, Località Germaneto, 88100 Catanzaro, Italy

**Keywords:** next-generation sequencing (NGS), hereditary cancer predisposition syndromes (HCPS), breast cancer (BC), genetic testing, pathogenic variants (PVs), breast and ovarian analysis of disease incidence and carrier estimation algorithm (BOADICEA)

## Abstract

Germline pathogenic variants (PVs) in oncogenes and tumour suppressor genes are responsible for 5 to 10% of all diagnosed cancers, which are commonly known as hereditary cancer predisposition syndromes (HCPS). A total of 104 individuals at high risk of HCPS were selected by genetic counselling for genetic testing in the past 2 years. Most of them were subjects having a personal and family history of breast cancer (BC) selected according to current established criteria. Genes analysis involved in HCPS was assessed by next-generation sequencing (NGS) using a custom cancer panel with high- and moderate-risk susceptibility genes. Germline PVs were identified in 17 of 104 individuals (16.3%) analysed, while variants of uncertain significance (VUS) were identified in 21/104 (20.2%) cases. Concerning the germline PVs distribution among the 13 BC individuals with positive findings, 8/13 (61.5%) were in the *BRCA1/2* genes, whereas 5/13 (38.4%) were in other high- or moderate-risk genes including *PALB2*, *TP53*, *ATM* and *CHEK2*. NGS genetic testing showed that 6/13 (46.1%) of the PVs observed in BC patients were detected in triple-negative BC. Interestingly, the likelihood of carrying the PVs in the moderate-to-high-risk genes calculated by the cancer risk model BOADICEA was significantly higher in pathogenic variant carriers than in negative subjects. Collectively, this study shows that multigene panel testing can offer an effective diagnostic approach for patients at high risk of hereditary cancers.

## 1. Introduction

Currently, inherited germline pathogenic variants in oncogenes and tumour suppressor genes are responsible for a small minority of cancers, around 5 to 10% of all diagnosed cancer cases, which are referred to as hereditary cancer predisposition syndrome (HCPS) [1]. More than 200 HCPS types and the associated genes have been described, which are normally driven by the presence of pathogenic variants in only one gene which confers an augmented risk of developing tumours at an early age in the affected individuals [2]. The majority of HCPS exhibit an autosomal dominant inheritance and include hereditary breast and ovarian cancer syndrome (HBOC), Lynch syndrome, Li–Fraumeni syndrome (LFS) and some others [3]. Inherited cancer susceptibility is suspected in a subject in which there is an earlier age of cancer diagnosis, several generations affected and the same cancer type on the same family side. In addition, the presence of two or more relatives with the same tumour and individuals with multiple primary tumours (MTP) indicates patients suspected of having HCPS [3].

In the past, genetic testing was based only on the high-penetrance genes such as *BRCA1* and *BRCA2*, which account for around 12 to 15% of ovarian cancers (OC) and 3 to 5% of breast cancers (BC) in most populations worldwide [4]. In the last years, it has been observed that HCPS, such as BC and OC, endometrial, gastric and colon cancers, are also associated with other genes such as *PALB2*, *MLH1*, *MSH2*, *PMS1*, *PMS2, MSH6, TP53*, *CDH1*, *SKT11* and *PTEN* [4].

BC is the most common type of cancer among women worldwide, accounting for 25% of the total number of new cancer cases. HBOC was known for decades to be caused by pathogenic variants in the *BRCA1* and *BRCA2* genes and is characterized by an increased risk of early-onset BC, male BC, epithelial OC, multiple BC and Fallopian tube cancer. However, prostate cancers (PC), melanoma and pancreatic cancer are also more common in subjects with HBOC [5]. Next-Generation Sequencing (NGS) studies showed that HBOC predisposition is linked to many genes, such as those with high penetrance, including, besides the above-mentioned *BRCA1* and *BRCA2*, also *TP53*, *PTEN*, *STK11*, *CDH1* and those with moderate or low penetrance including *ATM, CHEK2*, *PALB2*, *BRIP1*, *BARD1*, *RAD51C*, *RAD51D*, *NF1, NBN* and mismatch repair (MMR) genes [6]. In the current clinical practice, NGS approaches using a cancer panel with high- and moderate-risk susceptibility genes are commonly utilised for the identification of subjects with the HCPS [1].

Genetic counselling is an essential clinical activity based on the collection of individuals’ personal and family health history aiming to the identification of subjects who can gain benefit from the testing. In the context of the Italian national health system called “*Servizio Sanitario Nazionale*” (SSN), individuals belonging to specific HCPS, as well as their families, receive genetic counselling according to the Italian AIOM guidelines, which are similar to the internationally established guidelines within the framework of the national comprehensive cancer network (NCCN).

This study aimed to evaluate the frequency and the spectrum of the germline pathogenic variants in a cohort of 104 patients who underwent genetic counselling for suspected HCPS by using an NGS panel of high- and moderate-risk alleles. In addition, the likelihood of carrying pathogenic variants in the moderate-to-high-risk genes has been calculated for each HBOC and prostate cancer patient by the cancer risk model BOADICEA during genetic counselling, and the results were correlated with genetic testing to evaluate if there was a concordance between the precalculated risk score and the presence of PVs. The ability to distinguish HPCS from sporadic cancers that develop in individuals who have inherited a germline pathogenic variant is very useful in cancer surveillance and prevention. Determining the rate and the spectrum of germline PVs in the clinical population with cancer is important for promoting genetic counselling and testing. This study also evaluates if the cancer risk model BOADICEA can improve the selection of HCPS patients suitable for genetic testing.

## 2. Materials and Methods

### 2.1. Patients and Enrolling Criteria

A total of 104 patients were selected after genetic counselling at the Medical Genetics Unit (Mater Domini University Hospital at Catanzaro) between September 2019 and January 2022 for the NGS genetic test according to the family and personal criteria established by the National Comprehensive Cancer Network (NCCN) and the *Associazione Italiana di oncologia Medica* [7,8]. Genetic counselling was performed to evaluate the patient’s cancer history (clinical diagnosis, age of first cancer, histological stage) and family history of cancer (number of affected relatives).

The inclusion criteria for the NGS genetic tests were: (1) women with BC and OC; males with BC; women with triple-negative breast cancer (TNBC)<60 years; women with BC < 36 years; women with bilateral BC < 50 years; not mucinous and not borderline OC at any age; metastatic pancreatic adenocarcinoma; metastatic prostatic carcinoma. (2) Personal history of breast cancer diagnosed < 50 years and at least one first-degree relative with nonmucinous and nonborderline OC at any age; BC < 50 years; male BC; bilateral BC; metastatic pancreatic adenocarcinoma and metastatic prostatic carcinoma. (3) Personal history of BC > 50 years and family history of breast, ovarian cancer, metastatic prostatic carcinoma and metastatic pancreatic adenocarcinoma in 2 or more first-degree relatives (one of which in the first degree with the proband). (4) Presence of personal and family history that did not meet the AIOM criteria. (5) Patients that were not affected by tumours described by AIOM criteria. Based on biomarker expression, BC was categorised as Luminal A (ER+ and PgR+ and HER2-, Ki-67 low), Luminal B HER2- (ER+ and PgR+, HER2-, Ki-67 high), Luminal B HER2+ (ER+ and PgR +, HER2+, any Ki-67), HER2+ (ER- and PgR-, HER2+) and triple-negative (TN) (ER-, PgR- and HER2-) [9].

In addition to the selection criteria for the enrolled patients described above, the cancer risk model called Breast and Ovarian Analysis of Disease Incidence and Carrier Estimation Algorithm (BOADICEA) was used for the HBOC and prostate cancer patients [10] to estimate the likelihood of carrying *BRCA1*, *BRCA2*, *PALB2*, *CHEK2, ATM, BARD1, RAD51D, RAD51C* and *BRIP1* pathogenic variants (PVs). Other specific criteria for HPCS were used for patients with multiple cancer types, metastatic pancreatic adenocarcinoma diagnosed at any age and patients with pancreatic cancer or kidney cancer having a family history of cancer.

### 2.2. Genetic Testing: DNA Extraction and NGS

Genomic DNAs from patients were extracted from blood samples after signing informed consent forms using the NLM DNA extraction kit (Nuclear Laser Medicine) as previously described [11,12,13]. We designed two Ion Ampliseq On-Demand panels to explore, using NGS, the mutational status of the most frequently altered genes in HCPS. The panel includes *BRCA1*, *BRCA2*, *ATM*, *PALB2*, *TP53, CHEK2*, *MLH1*, *MSH2*, *MSH6* and *PMS2* (Thermo Fisher Scientific, Waltham, MA, USA) covering the full coding exons plus padding regions of the above-described genes. Libraries were constructed and purified on the Ion Chef Instrument according to the Ampliseq manufacturer’s instructions. Subsequently, libraries were sequenced by the Ion GeneStudio S5 System (Thermo Fisher Scientific, Waltham, MA, USA).

### 2.3. Sanger Sequencing

Genomic DNAs were amplified by PCR using the forward and reverse primer binding to the selected exons of *BRCA1*, *BRCA2*, *ATM*, *PALB2*, *TP53*, *CHEK2*, *MLH1*, *MSH2*, *MSH6* and *PMS2* genes. Amplicons were bidirectionally sequenced using Big Dye Terminator 1.1 on a SeqStudio Genetic Analyzer (Thermo Fisher Scientific, Waltham, MA, USA).

### 2.4. Variant Analysis (Classification): Germline Calling Variants and Filtering

Only germline variants with an allele frequency <0.01 based on allele frequencies found in GnomAD were retained for further investigation. Sequence variation databases such as ClinVar [14] and LOVD [15] were used to classify variants already reported, and when no data was available, the variant was classified following the American College of Medical Genetics (ACMG) criteria [16]. VUS variants were also classified using the semiquantitative, hierarchical evidence-based rules for the locus interpretation (Sherlock) method [17]. In addition, for VUS variants, computational prediction tools were used to predict the effect of the amino acid substitution on the protein function and structure.

### 2.5. Statistics

Differences between groups were assessed by the Mann–Whitney test (GraphPad Prism 9, GraphPad Software Inc., San Diego, CA, USA) at ** *p* < 0.01.

## 3. Results

### 3.1. Genetic Counselling and Clinical Features of Cancer Patients

**Figure 1:**
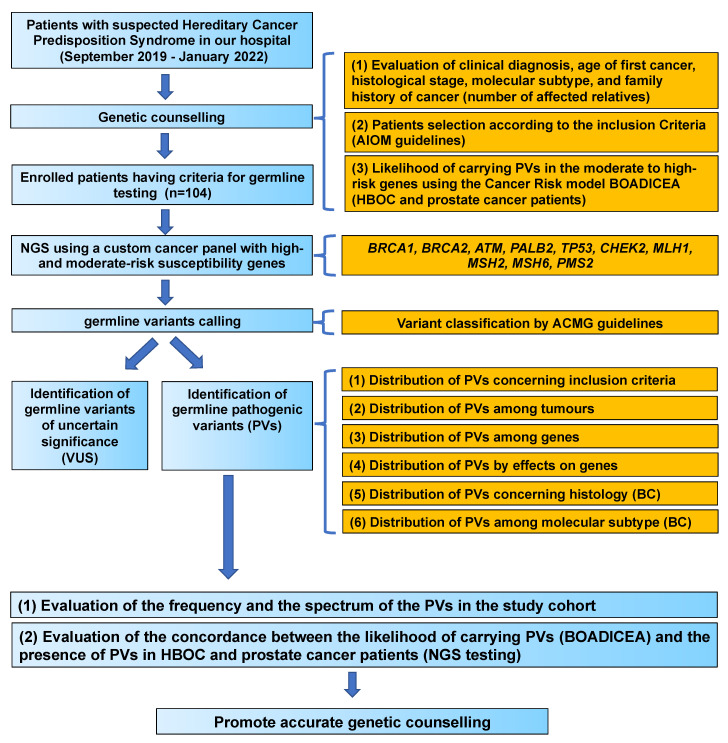
Study flow chart. PVs, pathogenic variants; VUS, variants of uncertain significance; BC, breast cancer; HBOC, hereditary breast and ovarian cancer syndrome.

The study flow chart is reported in Figure 1. Between September 2019 and January 2022, 104 patients were selected after genetic counselling for genetic tests according to the family and personal criteria established by the AIOM criteria based on the recommendations of the National Comprehensive Cancer Network (NCCN) [7]. Genetic counselling was performed to evaluate the patient’s cancer history, including clinical diagnosis, age of first cancer, histological stage, molecular subtype and family history of cancer (number of affected relatives) (Figure 1).

**Figure 2:**
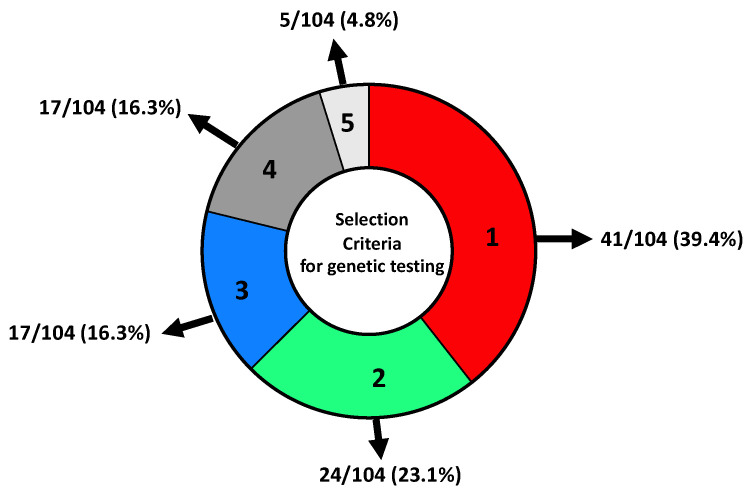
Distribution of patients selected for NGS genetic testing concerning inclusion criteria. (1) Women with BC and OC; males with BC; women with triple-negative breast cancer (TNBC) < 60 years; women with BC < 36 years; women with bilateral BC < 50 years; not mucinous and not borderline OC at any age; metastatic pancreatic adenocarcinoma; metastatic prostatic carcinoma. (2) Personal history of breast cancer diagnosed < 50 years and at least one first-degree relative with nonmucinous and nonborderline OC at any age; BC < 50 years; male BC; bilateral BC; metastatic pancreatic adenocarcinoma and metastatic prostatic carcinoma. (3) Personal history of BC > 50 years and family history of breast, ovarian cancer, metastatic prostatic carcinoma and metastatic pancreatic adenocarcinoma in 2 or more first-degree relatives (one of which in the first degree with the proband). (4) Presence of personal and family history that did not meet AIOM criteria. (5) Patients that were not affected by tumours described by AIOM criteria.

A total of 41/104 (39.4%) patients were selected for genetic testing following criteria 1 (Figure 2, see Materials and Methods for details), 24/104 (23.1%) patients were selected for genetic testing following criteria 2 and 17/104 (16.3%) following criteria 3. In addition, 17/104 (16.3%) patients did not meet the AIOM criteria but had personal and family history and were considered borderline, and 5/104 (4.8%) patients were selected using other criteria since they were not affected by tumours described in the AIOM criteria, such as uterine cancer, colon and LFS (Figure 2).

**Figure 3:**
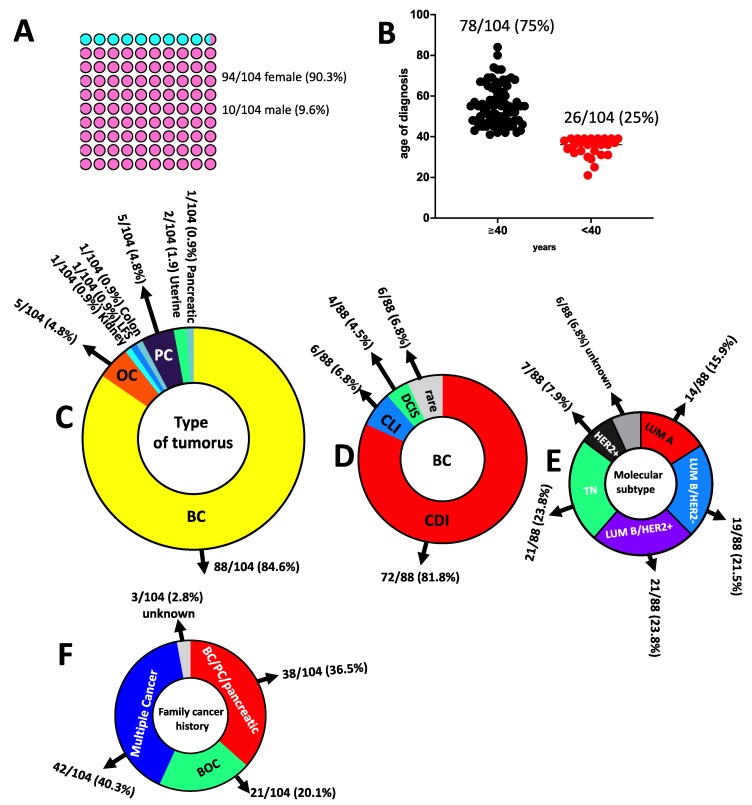
Characteristics of study participants. (**A**) Sex distribution. (**B**) Age of diagnosis. (**C**) Type of tumours. (**D**) BC histology. (**E**) BC molecular subtype. (**F**) Family cancer history; BC: breast cancer; BOC: breast and ovarian cancer; PC: prostate cancer; OC: ovarian cancer; TN: triple-negative; CDI: invasive ductal carcinoma; CLI: invasive lobular carcinoma; DCIS: ductal carcinoma in situ; LUM: luminal; LFS: Li–Fraumeni syndrome.

The study cohort included a total of 104 patients (94 females and 10 males; mean age of diagnosis 50.2, range 21–84 years, Figure 3A) with a clinical suspicion of hereditary cancer predisposition syndromes (HCPS) based on individual and family cancer history. A total of 26/104 (25%) patients were diagnosed with cancers before the age of 40 years, whereas 78/104 (75%) were diagnosed after 40 years of age (Figure 3B). At the first diagnosis, eighty-eight had breast cancer (BC), five had ovarian cancer (OC), five had prostate cancer (PC), one had colon cancer, one had pancreatic cancer, one had LFS, one had kidney cancer and two had uterine cancer (Figure 3C). Considering the 88 BC patients, 86 were monolateral and 2 were bilateral. Relating to BC histology, 72/88 (81.8%) had invasive ductal carcinoma (CDI), 4/88 (4.5%) had ductal carcinoma in situ (DCIS), 6/88 (6.8%) had invasive lobular carcinoma (CLI) and 6/88 (6.8%) had a rare BC histotype (Figure 3D). Among the 88 BC, the distribution molecular subtypes involved 14 (15.9%) Luminal A, 19 (21.5%) Luminal B-HER2-, 21 (23.8%) Luminal B-HER2+, 7 (7.9%) HER2+ and 21 (23.8%) TN (triple-negative) BC and 6 unknown molecular subtypes (6.8%) (Figure 3E). Among patients, 38/104 (36.5%) had a family history of BC, PC or pancreatic cancer, 21/104 (20.1%) had a history of BC and OC (HBOC), 3/104 (2.8%) had unknown family history whereas 42/104 (40.3%) were patients with a family history of multiple cancers (Figure 3F).

The detailed information for each enrolled patient, including patient ID, age, sex, age of diagnosis, type of cancer, histological grade, molecular subtype, cancer onset, the occurrence of multiple tumours and the presence of affected first- and second-degree relatives, and BOADICEA scores are listed in Supplementary Table S1.

### 3.2. Likelihood of Carrying PVs in the Moderate-to-High-Risk Genes Calculated by the Cancer Risk Model BOADICEA in HBOC and Prostate Cancer Patients

Currently, several cancer risk models are used to predict the risks of developing HBOC and prostate cancer and to calculate the likelihood of carrying PVs in the moderate-to-high-risk genes. In this context, BOADICEA is considered the most accurate algorithm able to predict combined *BRCA1*/2 pathogenic variants with respect to other predictor models such as BRCAPRO, Penn II and Myriad [18]. In addition, the last V5 version of BOADICEA incorporates the effects of pathogenic variants (PVs), not only in *BRCA1* and *BRCA2* genes, but also in *PALB2, CHEK2*, *ATM* and *BARD1* for the breast cancer model and *RAD51D, RAD51C* and *BRIP1* for the ovarian cancer model [19]. The likelihood of carrying PVs for each patient is calculated by the BOADICEA model based on personal and family cancer history, mammographic density, histology, molecular subtype, hormonal risk factors and lifestyle.

**Figure 4:**
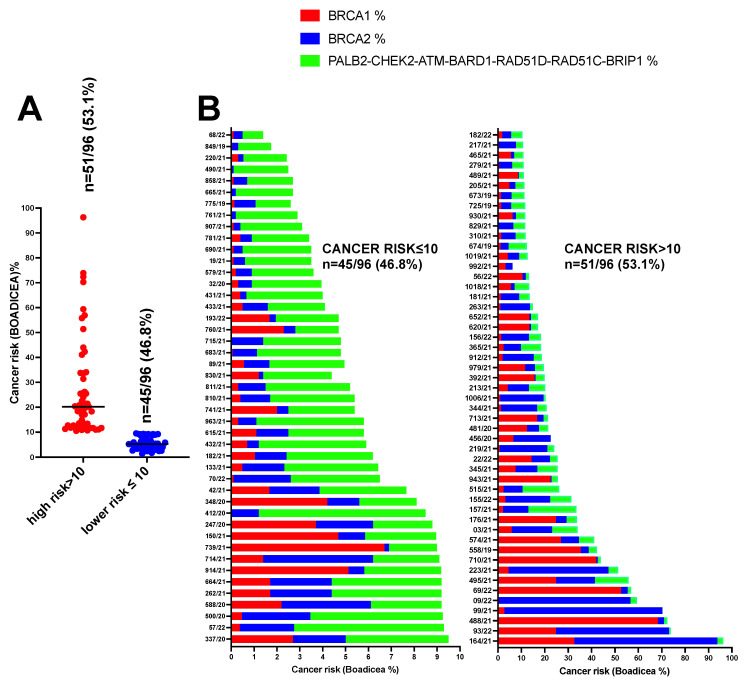
Likelihood of carrying PVs in the moderate-to-high-risk genes in eighty-eight BC, five OC and three PC (n = 96) patients using a 10% pretest probability threshold. (**A**) Number of patients having BOADICEA >10% and ≤10%. (**B**) Patient’s risk in the well-known high-penetrance alleles *BRCA1* and *BRCA2* with respect to other moderate-penetrance alleles including *PALB2*, *CHEK2, ATM, BARD1, RAD51D, RAD51C* and *BRIP1*.

Therefore, the BOADICEA prediction model was used to calculate the likelihood of carrying pathogenic variants in the moderate-to-high-risk genes in eighty-eight BC, five OC and three PC patients (n = 96) using a 10% pretest probability threshold. A total of 51/96 (53.1%) patients having BOADICEA >10% probability were classified as high risk, whereas 45/96 (46.8%) patients with a percentage ≤10% were considered as low risk to be carriers of pathogenic variants in *BRCA1*, *BRCA2*, *PALB2, CHEK2, ATM*, *BARD1*, *RAD51D, RAD51C* and *BRIP1* genes (Figure 4A). For each patient, we also showed the risk in the well-known high-penetrance cancer risk alleles *BRCA1* and *BRCA2* with respect to other moderate-penetrance alleles including *PALB2*, *CHEK2*, *ATM, BARD1, RAD51D, RAD51C* and *BRIP1* (Figure 4B).

### 3.3. Genetic Testing and Variants Distribution

**Figure 5:**
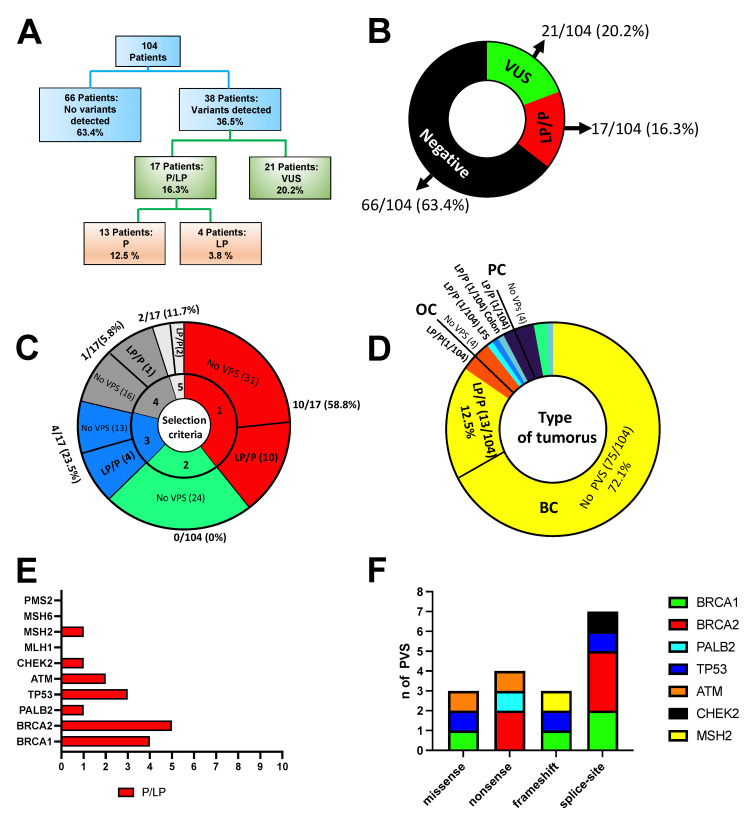
(**A**) Overall results of NGS panel testing. Importantly, the VUS rate does not include VUS detected in patients with P/LP variants. (**B**) Outcomes of panel testing for the 104 individuals tested. (**C**) Distribution of PVs concerning enrolling criteria. (**D**) Distribution of PVs among tumours. (**E**) Distribution of PVs among genes. (**F**) Distribution of 17 pathogenic variants by effect.

Gene panel sequencing yielded germline uncommon variants in 38 of 104 individuals (36.5%). No variants were detected in 66 individuals (63.4%), while 21 (20.2%) of the subjects had at least one VUS, and 17 (16.3%) had variants which were P/LP (Figure 5A, B). Pathogenic variants (P) were detected in 13 patients (12.5%), whereas likely pathogenic variants (LP) were found in 4 (3.8%) of the subjects enrolled in this study.

Of the 17 P/LP variants detected by the NGS panel testing (Table 1), ten fulfilled AIOM criteria 1 (58.8%), four fulfilled criteria 3 (23.5%), one did not fulfil criteria (PALB2:c.1451T>A) and two patients were not affected by tumours described by AIOM, and thus were included considering other specific criteria, including one LFS and one colon cancer (11.7%, Figure 5C). In the colon cancer patient, we found the *MSH2* PV (c.1204del), whereas *TP53* PV (c.645delT) was found in a patient with LFS. A total of 82.3% of patients who received positive results in this study fulfilled the AIOM testing criteria. Thirteen pathogenic variants (PVs) (12.5%) were found in patients with BC, one patient with OC (0.9%), one with colon cancer (0.9%), one with LFS (0.9%) and one with prostate cancer (0.9%) (Figure 5D). In total, four (3.8%) VPs were in *BRCA1*, five were in *BRCA2* (4.8%), one in *PALB2* (0.96%), three in *TP53* (2.8%), two in *ATM* (1.92%), one in *CHEK2* (0.96%) and one in *MHS2* (0.96%) (Figure 5E).

Among the BC patients, 8/13 (61.5%) VPs were in the *BRCA1/2* genes, whereas 5/13 (38.4%) were in other high- and moderate-risk genes, including *PALB2* (c.1551T>G)*, TP53* (c.451C>G and c.376-1G>A), *ATM* (c.6100C>T) and *CHEK2* (c.846+1G>C).

Three of seventeen (17.6%) identified LP/P pathogenic variants were missense, four nonsense (23.5%) variants, three frameshift variants (17.6%) and seven splice-site variants (41.1%). The distribution of variants by effect is shown in Figure 5F. The complete list of pathogenic and likely pathogenic variants identified in this study, and the further details, including HGVS nomenclature, allelic frequencies, variant type, Clinvar classification and tumour type, is shown in Table 1.

**Figure 6:**
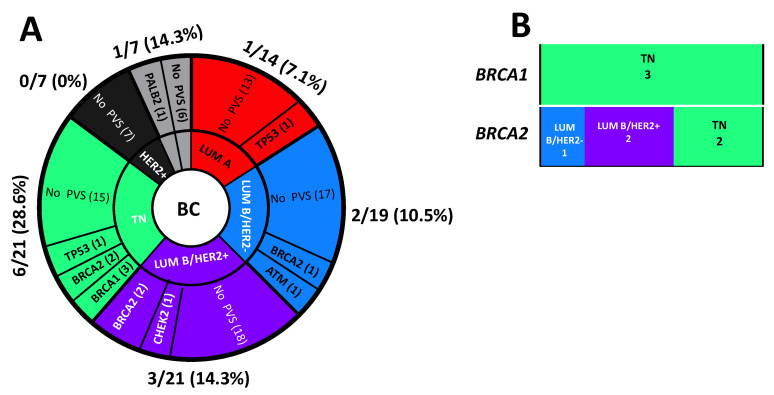
(**A**) Distribution of molecular subtypes in the study cohort. (**B**) Prevalence of molecular subtypes in positive *BRCA1*- and *BRCA2*- breast cancer patients.

Interestingly, 6 PVs out of 13 (46.1%) observed in the BC patients were detected in triple-negative BC. In particular, 6 of 21 (28.6%) triple-negative BC patients, 3 of 21 (14.3%) Luminal B/HER2+ BC patients, 2 of 19 (10.5%) Luminal B/HER2- BC patients, 1 of 14 (7.1%) Luminal A BC patients and 1 of 7 (14.3%) patients with unknown molecular subtype were carriers of PVs (Figure 6A).

Among the BC patients positive for *BRCA1*-, three (100%) had a triple-negative BC whereas, among those positive for *BRCA2*- tumours, two were triple-negative BC (40%), one was Luminal B/HER2- (20%) and two were Luminal B/HER2+ (40%, Figure 6B).

**Table 1:** Pathogenic variants identified in this study.

n.	Patient ID	Variant (HGVS) GRCh37	Gene with Variant	dpSNP (Varsome Link)	Type of Variant	MAF gnomAD%	Clinvar Classification	Ref	Type of Cancer
**1**	**558/19**	chr17:g.41258504A>C c.181T>G (p.Cys61Gly)	*BRCA1*	rs28897672	missense	0.0031	Pathogenic	[20]	Ovarian cancer
**2**	**673/19**	chr11:g.108186742C>T c.6100C>T (p.Arg2034Ter)	*ATM*	rs532480170	nonsense	0.0004	Pathogenic	[21,22]	Breast cancer
**3**	**764/19**	chr17:g.7578204del c.645delT (p.Ser215ArgfsTer32)	*TP53*	NR	frameshift	NR	Pathogenic	[11]	Li–Fraumeni
**4**	**775/19**	chr11:g.108236087G>A c.9023G>A (p.Arg3008His)	*ATM*	rs587781894	missense	NR	Likely pathogenic	[23,24,25,26,27]	Prostate cancer
**5**	**99/21**	chr22:g.29105993C>A c.846+1G>C	*CHEK2*	rs864622149	splice-site	NR	Likely pathogenic	[28,29]	Breast cancer
**6**	**164/21**	chr17:g.41267741A>G c.134+2T>C	*BRCA1*	rs80358131	splice-site	NR	Pathogenic	[20,30]	Breast cancer
**7**	**223/21**	chr13:g.32944695G>A c.8487+1G>A	*BRCA2*	rs81002798	splice-site	NR	Pathogenic	[31,32,33]	Breast cancer
**8**	**279/21**	chr13:g.32921033G>A c.7007G>A (p.Arg2336His)	*BRCA2*	rs28897743	splice-site (*)	NR	Pathogenic	[34,35,36]	Breast cancer
**9**	**365/21**	chr13:g.32907285T>G c.1670T>G (p.Leu557Ter)	*BRCA2*	rs80358452	nonsense	NR	Pathogenic	[37,38,39]	Breast cancer
**10**	**432/21**	chr16:g.23646416A>C c.1451T>G (p.Leu484Ter)	*PALB2*	rs786203714	nonsense	NR	Pathogenic	[40,41,42,43]	Breast cancer
**11**	**488/21**	chr17:g.7578479G>C c.451C>G (p.Pro151Ala)	*TP53*	NR	missense	NR	Likely pathogenic	[44,45]	Breast cancer
**12**	**713/21**	chr13:g.32907526T>A c.1909+2T>A	*BRCA2*	rs876658577	splice-site	NR	Likely pathogenic	NR	Breast cancer
**13**	**812/21**	Chr2:g.47429869del c.1204del (p.Gln402LysfsTer10)	*MSH2*	rs63751413	frameshift	NR	Pathogenic	[46]	Colon cancer
**14**	**930/21**	chr13:g.32333148T>G c.1670T>G (p.Leu557Ter)	*BRCA2*	rs80358452	nonsense	NR	Pathogenic	[37]	Breast cancer
**15**	**943/21**	chr17:g.41223012_41223030del c.4964_4982del p.(Ser1655TyrfsTer16)	*BRCA1*	rs1555580678	frameshift	NR	Pathogenic	NR	Breast cancer
**16**	**22/22**	chr17:g.7578555C>T c.376-1G>A	*TP53*	rs868137297	splice-site	0.00000657	Pathogenic	[47]	Breast cancer
**17**	**69/22**	chr17:g.431157724A>C c.134+2T>G	*BRCA1*	rs80358131	splice-site	NR	Pathogenic	[48]	Breast cancer

Minor allele frequency (MAF), Clinical variation database [14]; Human Genome Variation Society [49]; nonreported (NR). (*), This missense variant affects splicing [50].

### 3.4. VUS, Variants Classification by ACMG Guidelines and Reclassification by SHERLOC Framework

In total, 24 VUS were found in 21 patients, since 2 of these patients (281/21 and 279/21) had more than one VUS. A detailed list of VUS variants identified in this study is provided in Table 2. In addition, six further VUS were found in patients (775/19, 99/21, 164/21, 432/21 and 943/21) for which other LP or P variants were identified (Supplementary Table S1). Considering a distinction for pathology, among the sixteen BC patients with no other LP/P variants, three harboured VUS in the *BRCA1* gene (18.7%), four patients harboured VUS in the *ATM* (25%), five VUS in *CHEK2* (31.2%), one in *MSH6* gene (6.2%), one in *MLH1* (6.2%) and two in *PMS2* (12.5%) (Table 2). As regards the germline variant type, most were missense variants (n = 19), and the remaining were one 5′-UTR (*CHEK2*:c.-4C>T) and one splice variant (*MLH1*:c.678-4A>G).

All VUS variants identified in this study were reclassified by the Sherlock interpretation framework [17], and the results showed that all (100%) variants did not change from their previous VUS classification (Supplementary Table S2). Therefore, the clinical significance of the 21 VUSs described in this study remains unclear, since there is not sufficient evidence to associate them with a pathogenicity condition.

**Table 2:** VUS variants found in HCPS patients.

n.	Patient ID	Variant (HGVS) GRCh37	Gene with Variant	dpSNP	Type of Variant	MAF gnomAD%	Clinvar Classification	Ref	Type of Cancer
**1**	**674/19**	chr11:g.108201108T>G c.7475T>G (p.Leu2492Arg)	*ATM*	rs56399857	missense	0.0099	VUS	[51,52]	Breast cancer
**2**	**348/20**	chr11:g.108150289C>T c.3356C>T (p.Ala1119Val)	*ATM*	rs778882461	missense	0.0039	VUS	NR	Breast cancer
**3**	**704/20**	chr11:g.108200949T>C c.7316T>C (p.Val2439Ala)	*ATM*	rs776266049	missense	0.0004	VUS	[53,54,55]	Prostate cancer
**4**	**87/21**	chr16: g.23652442C>T c.37G>A (p.Glu13Lys)	*PALB2*	rs373287455	missense	0.0004	VUS	[41,52,56,57,58,59]	Kidney cancer
**5**	**133/21**	chr22:g.29095923A>G c.911T>C (p.Met304Thr)	*CHEK2*	rs587782033	missense	NR	VUS	[60,61,62,63,64]	Breast cancer
**6**	**150/21**	chr17:g.41246204G>C c.1344C>G (p.His448Gln)	*BRCA1*	NR	missense	NR	VUS	NR	Breast cancer
**7**	**182/21**	chr17:g.41203100G>T c.5312C>A (p.Pro1771His)	*BRCA1*	NR	missense	NR	VUS	[20]	Breast cancer
**8**	**262/21**	chr2:g.48028063A>G c.2941A>G (p.Ile981Val)	*MSH6*	rs730881799	missense	NR	VUS	[65]	Breast cancer
**9**	**282/21**	chr22: g.29091178C>A c.1312G>T (p.Asp438Tyr) chr3:g.37055919A>G c.678-4A>G chr2:g.48026120C>T c.998C>T (p.Thr333Ile)	*CHEK2* *MLH1* *MSH6*	rs2000508 83 rs766711342 rs587781983	missense splice-site missense	0.039 0.0012 0.0032	VUS VUS VUS	[66,67,68,69,70,71,72,73]	Pancreatic cancer
**10**	**310/21**	chr16:g.23647304G>C c.563C>G (p.Ala188Gly)	*PALB2*	rs587781975	missense	0.0011	VUS	NR	Breast cancer
**11**	**344/21**	chr22:g.29091178C>A c.1312G>T (p.Asp438Tyr)	*CHEK2*	rs200050883	missense	0.039	VUS	[66,67,68]	Breast cancer
**12**	**465/21**	chr11:g.108142010A>G c.2954A>G (p.Asp985Gly)	*ATM*	rs864622159	missense	0.0004	VUS	NR	Breast cancer
**13**	**489/21**	chr2:g.48026433-48026434delinsGC c.1311_ 1312delinsGC (p.437_438delinsGlnLeu)	*MSH6*	NR	missense		VUS	[74]	Ovarian cancer
**14**	**620/21**	chr22:g.28711914C>G c.787G>C (p.Glu263Gln)	*CHEK2*	rs730881686	missense	0.00000796	VUS	[60,75]	Breast cancer
**15**	**665/21**	chr22:g.29091797G>A c.1160C>T (p.Thr387Ile)	*CHEK2*	rs587780168	missense	0.00000398	VUS	[76,77]	Breast cancer
**16**	**760/21**	chr7:g.6043346C>A c.328G>T (p.Ala110Ser)	*PMS2*	rs767775907	missense	0.0000169	VUS	[78]	Breast cancer
**17**	**761/21**	chr7:g.6043346C>A c.328G>T (p.Ala110Ser)	*PMS2*	rs767775907	missense	0.0000169	VUS	[78]	Breast cancer
**18**	**979/21**	chr22:g.29130713G>A c.-4C>T chr3:g.37092003C>G c.2130C>G (p.Asn710Lys)	*CHEK2* *MLH1*	rs3749381 48 rs7491000 96	5′-UTR variant missense	0.0000573 0.00000398	VUS VUS	[79] NR	Breast cancer
**19**	**1006/21**	chr11:g.108300949T>C c.7316T>C (p.Val2439Ala)	*ATM*	rs776266049	missense	0.00000398	VUS	[53]	Breast cancer
**20**	**68/22**	chr11:g.108224555 c.8734A>G (p.Arg2912Gly)	*ATM*	rs376676328	missense	0.000219	VUS	[80]	Prostate cancer
**21**	**156/22**	chr17:g.41246298T>C c.1250A>G (p.Asn417Ser)	BRCA1	rs80357113	missense	NR	VUS	[81]	Breast Cancer

Minor allele frequency (MAF), Clinical variation database [14]; Human Genome Variation Society [49]; nonreported (NR).

### 3.5. Likelihood of Carrying PVs in LP/P Variant-Positive HBOC and Prostate Cancer Patients versus Negative or Patients Carrying VUS

**Figure 7:**
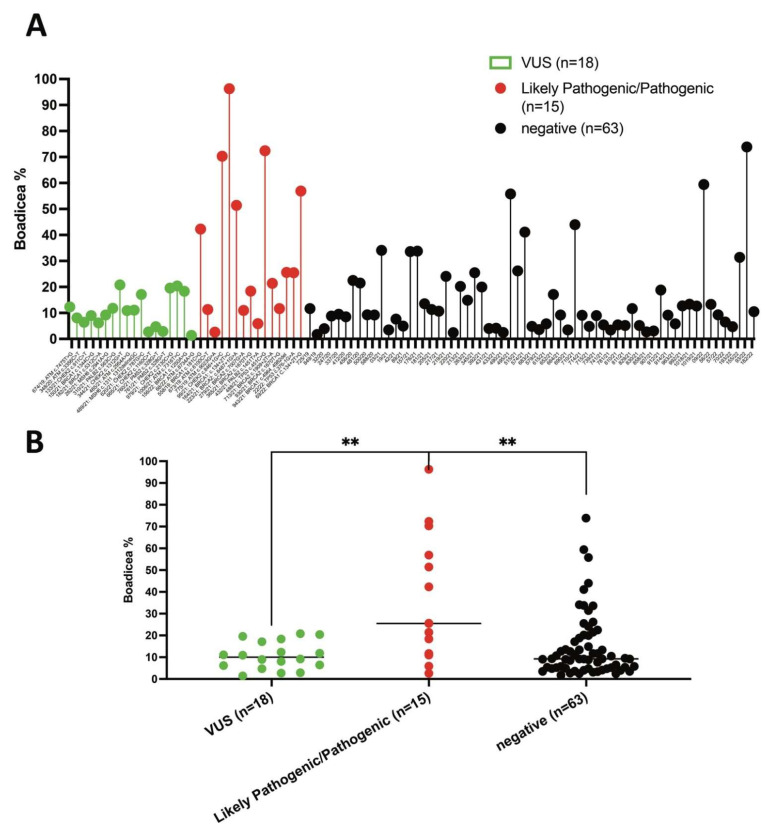
BOADICEA scores in LP/P variant-positive HBOC and prostate cancer patients versus negative and patients carrying VUS. (**A**) Distribution of total % BOADICEA score (likelihood of carrying PVs, sum of *BRCA1*, *BRCA2*, *PALB2, CHEK2, ATM*, *BARD1*, *RAD51D, RAD51C* and *BRIP1*) for each patient. (**B**) BOADICEA scores between groups of patients depending on variant status (VUS, LP/P and negative). Differences between groups were assessed by the Mann–Whitney test; ** *p* < 0.01.

Considering the likelihood of carrying PVs in the moderate-to-high-risk genes in HBOC and prostate cancer patients, calculated by the BOADICEA model during genetic counselling with respect to the results of genetic testing, patients in the cohort were divided into three subgroups: (1) patients carrying VUS, (2) patients with LP/P pathogenic variants and (3) patients with no detected variants (Figure 7A). Interestingly, the BOADICEA score was significantly higher in the likely pathogenic/pathogenic variant-positive HBOC and prostate cancer patients versus the variant-negative individuals or HBOC patients carrying the VUS variants (Figure 7B). Although the study cohort was too limited, the ability of BOADICEA to predict *BRCA1* and *BRCA2* pathogenic variants seems better than the ability to predict pathogenic variants in other genes including *PALB2*, *CHEK2, ATM, BARD1, RAD51D, RAD51C* and *BRIP1* (Supplementary Figures S1 and S2).

## 4. Discussion

The current study performed NGS analyses by evaluating 10 known disease-causing genes for HCPS on 104 enrolled patients from South Italy having a strong personal and family history chosen after genetic counselling. The major number of PVs were found in *BRCA2* (n = 5), followed by *BRCA1* (n = 4), *TP53* (n = 3) and *ATM* (n = 2). *PALB2*, *CHEK2* and *MSH2* were found mutated in one patient only. Except for the PVs in *ATM* (c.9023G>A) found in the PC patient, *TP53* (c.645del) in the patient affected by LFS, *MSH2* (c.1204del) in the patient with colon cancer and *BRCA1* (c.181C>T) in the OC patient, all the remaining PVs were found in the BC patients (n = 13).

The *TP53* variant (c.645del) detected in patient number 764/19 was previously described in a Southern Italian family having an aggregated history of typical LFS cancers [11]. Germline *TP53* PVs are associated with a wide range of cancers, known collectively as LFS, which is characterised by a predisposition towards a broad spectrum of malignancy, including soft-tissue sarcomas, adrenocortical carcinomas, brain tumours, early-onset BC and leukaemias [11].

Among the 13 BC-positive patients, 8/13 (61.5%) were in the *BRCA1/2* genes. The PVs identified in *BRCA1* were c.134+2T>G, c.134+2T>C and c.4964_4982del, whereas in *BRCA2*, they were c.8487+1G>A, c.7007G>A, c.1670T>G and c.1909+2T>A. The deletion c.4964_4982del in *BRCA1*, also known as 5083del19, was reported in the BC/OC patients by Baudi et al. as a founder mutation in South Italy (Calabria) [74] and was detected in several subjects with BC and OC [74,82]. The nonsense variant c.1670T>G, also known as L557X in the *BRCA2*, was reported in several subjects affected with BC or OC [39,83]. Interestingly, the *BRCA2:* c.1670T>G was found in two patients in this study cohort and a large study of the Italian population detected this PV in four Calabrian patients [84].

A total of (5/13) 38.4% of BC patients harboured PVs in genes with moderate-to-high risk for BC, including *PALB2* (c.1451T>G), *TP53* (c.451C>G and c.376-1G>A)*, ATM* (c.6100C>T) and *CHEK2* (c.846+1G>C).

In addition to *BRCA1/2*, *PALB2* is the most important gene involved in BC susceptibility [85]. Moreover, *TP53*, a well-known gene involved in Li–Fraumeni syndrome (LFS), is another high-penetrance BC susceptibility gene [86]. Two BC cases (488/21 and 22/22) that did not fulfil the classic LFS criteria were found to have PVs in *TP53* (c.451C>G and c.376-1G>A). Our observations were consistent with that of a previous study in which some multiplex NGS panels for BC patients also detected several *TP53* PVs that did not fulfil the classic LFS criteria [86].

Together with the previously described genes, *ATM* is also currently included in the major part of NGS panels for BC, since it is considered a moderate-penetrance BC susceptibility gene. In addition, it was observed that the relative risk of BC in a patient carrying a pathogenic variant of *ATM* is increased more than three times compared with the general population [21]. However, it has been observed that some variants in the *ATM* gene can be associated with a different degree of BC risk than other variants in the same gene. For example, the presence of the c.7271T>G PVs in *ATM* is associated with a significantly increased risk for early-onset BC, but the association between other PVs in *ATM*, such as 5557G>A or ivs38-8T>C, still remained unclear [87,88]. Therefore, although some germline PVs in the *ATM* gene have been shown to have an increased risk for BC, the role of *ATM* in BC risk is not fully understood, since the penetrance of some PVs appears similar to that of *BRCA2* whereas others do not [88]. Although *CHEK2* VPs are rare in BC, they can potentially contribute to BC susceptibility, since some interactions between mutated *CHEK2* with other genes can be associated with BC development [29].

Interestingly, some PVs in *CHEK2* seem to confer a higher cancer risk than others [89].

Currently, several methods and tools were developed in clinical genetic counselling to estimate the likelihood that a subject is a carrier of a PV in the *BRCA1* or *BRCA2* [90]. Among these models, BOADICEA can be used in clinical practice to promote genetic counselling and increase the prevention and surveillance of BC development [90]. Importantly, the BOADICEA model is accepted by the Care Excellence and NIH in the UK, which recommends a mutation probability threshold of 10% to select patients for genetic testing of *BRCA1* and *BRCA2* [90]. In this study, the likelihood of carrying PVs in the moderate-to-high-risk genes in the HBOC and PC patients was evaluated by the BOADICEA model during genetic counselling. In the phase of genetic counselling, it should be assumed that the BOADICEA can estimate only the probability of carrying a PV in a subject and not the probability of detecting a PV [91]. To evaluate if the BOADICEA model can distinguish between carriers and noncarriers of PVs, the results of genetic tests were compared with the precalculated risks obtained for each patient during genetic counselling. Interestingly, this study shows that BOADICEA can distinguish between carriers and noncarriers of PVs, since the calculated score was significantly higher in the pathogenic variant-positive patients versus the variant-negative individuals with BC. However, we observed that some BC patients with high cancer risk BOADICEA scores resulted negatively for pathogenic variants (PVs). This could be because the detection of larger indels and exon-level copy number variants (CNVs) by NGS was not included in the workflow analysis of this study. In the current clinical practice, although only *BRCA1* and *BRCA2* are routinely evaluated in terms of large genomic rearrangements (LGRs), it has been observed that an important proportion of *PALB2 PVs* in BC subjects are LGRs [85]. Therefore, in addition to the *BRCA1* and *BRCA2* LRGs, *PALB2* LGRs should also be included in routine clinical genetic testing.

The main limitations of this study are the limited size of the study cohort and the fact that the cohort is enriched for BC. In addition, this study did not include the detection of large rearrangements such as CNVs in *BRCA1*/*2* or *PALB2*. Therefore, it cannot exclude that some patients of the study cohort at high risk of BC who were negative for single nucleotide variants (SNVs) and small insertions and deletions (Indels) are carriers of CNVs.

The purpose of this study in general is a description of the PVs found in the patients that were selected in our hospital by evaluating the extent and nature of PVs in the genes mainly implicated in HPCS. Another purpose of this study is to understand if our genetic counselling approach, combined with the multigene panel selected by us, has a clinical significance in hereditary cancer predisposition syndrome, especially in BC. Shortly, the trend is to use larger NGS panels for evaluating PVs in HPCS. However, although the addition of many moderate-to-low-risk genes into NGS panels could increase the diagnostic yield [92], this method can lead to complex findings, since the penetrance of particular germline variants of cancer-associated genes is yet to be defined.

The next step of this study will be the evaluation of CNVs in patients who were negative for SNV/indels by computational algorithms and their confirmation by orthogonal methods. In conclusion, this study shows that the multigene panel testing selected can offer an effective diagnostic approach for BC patients. In addition, the results of this study suggest that an accurate evaluation of the probability that the subject is a carrier of a germline PV in high-risk susceptibility genes is important to help counsellors to evaluate whether genetic testing is appropriate or not.

## Data Availability

The data can be requested by emailing the corresponding authors.

## Supplementary Materials

The following supporting information can be downloaded at: https://www.mdpi.com/article/10.3390/genes13071286/s1, Table S1: Detailed information for each enrolled patient, including patient ID, age, sex, age of diagnosis, type of cancer, histological grade, molecular subtype, cancer onset, the occurrence of multiple tumours and the presence of affected first- and second-degree relatives, and BOADICEA scores; Table S2: Interpretation of VUS variants using ACMG rules and semiquantitative rules by Sherloc [46]; Figure S1: Likelihood of carrying PVs in *BRCA1* and *BRCA2* positive patients; Figure S2: Likelihood of carrying PVs in non-*BRCA1/2* positive patients.

## References

[1] Tsaousis G.N., Papadopoulou E., Apessos A., Agiannitopoulos K., Pepe G., Kampouri S., Diamantopoulos N., Floros T., Iosifidou R., Katopodi O. (2019). Analysis of hereditary cancer syndromes by using a panel of genes: Novel and multiple pathogenic mutations. BMC Cancer.

[2] Ramírez-Calvo M., García-Casado Z., Fernández-Serra A., de Juan I., Palanca S., Oltra S., Soto J.L., Castillejo A., Barbera V.M., Juan-Fita M.J. (2019). Implementation of massive sequencing in the genetic diagnosis of hereditary cancer syndromes: Diagnostic performance in the Hereditary Cancer Programme of the Valencia Community (FamCan-NGS). Hered. Cancer Clin. Pract..

[3] Nagy R., Sweet K., Eng C. (2004). Highly penetrant hereditary cancer syndromes. Oncogene.

[4] Bhai P., Levy M.A., Rooney K., Carere D.A., Reilly J., Kerkhof J., Volodarsky M., Stuart A., Kadour M., Panabaker K. (2021). Page Title: Analysis of sequence and copy number variants in Canadian patient cohort with familial cancer syndromes using a unique next generation sequencing based approach. Front. Genet..

[5] Hampel H., Bennett R.L., Buchanan A., Pearlman R., Wiesner G.L. (2015). A practice guideline from the American College of Medical Genetics and Genomics and the National Society of Genetic Counselors: Referral indications for cancer predisposition assessment. Genet. Med..

[6] Angeli D., Salvi S., Tedaldi G. (2020). Genetic predisposition to breast and ovarian cancers: How many and which genes to test?. Int. J. Mol. Sci..

[7] Daly M.B., Pilarski R., Yurgelun M.B., Berry M.P., Buys S.S., Dickson P., Domchek S.M., Elkhanany A., Friedman S., Garber J.E. (2020). NCCN guidelines insights: Genetic/familial high-risk assessment: Breast, ovarian, and pancreatic, version 1.2020: Featured updates to the NCCN guidelines. J. Natl. Compr. Cancer Netw..

[8] AIOM Guidelines. https://www.aiom.it/raccomandazioni-per-limplementazione-del-test-brca-predittivo-e-preventivo-nei-tumori-della-mammella-dellovaio-del-pancreas-e-della-prostata/.

[9] Goldhirsch A., Winer E.P., Coates A., Gelber R., Piccart-Gebhart M., Thürlimann B., Senn H.-J., Albain K.S., André F., Bergh J. (2013). Personalizing the treatment of women with early breast cancer: Highlights of the St Gallen International Expert Consensus on the Primary Therapy of Early Breast Cancer 2013. Ann. Oncol..

[10] BODICEA. https://www.canrisk.org/canrisk_tool.

[11] Paduano F., Fabiani F., Colao E., Trapasso F., Perrotti N., Barbieri V., Baudi F., Iuliano R. (2021). Case Report: Identification of a Novel Pathogenic Germline TP53 Variant in a Family With Li-Fraumeni Syndrome. Front. Genet..

[12] Paduano F., Colao E., Grillone T., Vismara M.F.M., Amato R., Nisticò S., Mignogna C., Dastoli S., Fabiani F., Zucco R. (2021). A Familial Form of Epidermolysis Bullosa Simplex Associated with a Pathogenic Variant in KRT5. Genes.

[13] Paduano F., Colao E., Loddo S., Orlando V., Trapasso F., Novelli A., Perrotti N., Iuliano R. (2020). 7q35 Microdeletion and 15q13.3 and Xp22.33 Microduplications in a Patient with Severe Myoclonic Epilepsy, Microcephaly, Dysmorphisms, Severe Psychomotor Delay and Intellectual Disability. Genes.

[14] Clinvar. http://clinvar.com/.

[15] LOVD. https://www.lovd.nl/.

[16] Richards S., Aziz N., Bale S., Bick D., Das S., Gastier-Foster J., Grody W.W., Hegde M., Lyon E., Spector E. (2015). Standards and guidelines for the interpretation of sequence variants: A joint consensus recommendation of the American College of Medical Genetics and Genomics and the Association for Molecular Pathology. Genet. Med..

[17] Nykamp K., Anderson M., Powers M., Garcia J., Herrera B., Ho Y.-Y., Kobayashi Y., Patil N., Thusberg J., Westbrook M. (2017). Sherloc: A comprehensive refinement of the ACMG–AMP variant classification criteria. Genet. Med..

[18] Hung F.-H., Wang Y.A., Jian J.-W., Peng H.-P., Hsieh L.-L., Hung C.-F., Yang M.M., Yang A.-S. (2019). Evaluating BRCA mutation risk predictive models in a Chinese cohort in Taiwan. Sci. Rep..

[19] Lee A., Mavaddat N., Cunningham A.P., Carver T., Archer S., Walter F.M., Tischkowitz M., Roberts J., Usher-Smith J., Simard J. (2022). Enhancing the BOADICEA cancer risk prediction model to incorporate new data on RAD51C, RAD51D, BARD1, updates to tumour pathology and cancer incidences. medRxiv.

[20] Findlay G.M., Daza R.M., Martin B., Zhang M.D., Leith A.P., Gasperini M., Janizek J.D., Huang X., Starita L.M., Shendure J. (2018). Accurate classification of BRCA1 variants with saturation genome editing. Nature.

[21] Yang Z., Ouyang T., Li J., Wang T., Fan Z., Fan T., Lin B., Zhang J., Xie Y. (2019). Prevalence and characterization of ATM germline mutations in Chinese BRCA1/2-negative breast cancer patients. Breast Cancer Res. Treat..

[22] Perkins B.A., Caskey C.T., Brar P., Dec E., Karow D.S., Kahn A.M., Hou Y.C., Shah N., Boeldt D., Coughlin E. (2018). Precision medicine screening using whole-genome sequencing and advanced imaging to identify disease risk in adults. Proc. Natl. Acad. Sci. USA.

[23] Feliubadaló L., Moles-Fernández A., Santamariña-Pena M., Sánchez A.T., López-Novo A., Porras L.M., Blanco A., Capellá G., de la Hoya M., Molina I.J. (2021). A Collaborative Effort to Define Classification Criteria for ATM Variants in Hereditary Cancer Patients. Clin. Chem..

[24] Milanovic M., Houghton L.M., Menolfi D., Lee J.H., Yamamoto K., Li Y., Lee B.J., Xu J., Estes V.M., Wang D. (2021). The Cancer-Associated ATM R3008H Mutation Reveals the Link between ATM Activation and Its Exchange. Cancer Res..

[25] Ida K., Miyamoto T., Higuchi S., Takeuchi H., Yamada S., Ono M., Nishihara H., Shiozawa T. (2019). Effectiveness of a genetic test panel designed for gynecological cancer: An exploratory study. Med. Oncol..

[26] Gong R., He Y., Liu X.Y., Wang H.Y., Sun L.Y., Yang X.H., Li B., Cao X.K., Ye Z.L., Kong L.H. (2019). Mutation spectrum of germline cancer susceptibility genes among unselected Chinese colorectal cancer patients. Cancer Manag. Res..

[27] Nielsen S.M., De Simone L.M., Olopade O.I. (2018). Cancer Susceptibility Genetic Testing in a High-Risk Cohort of Urban Ashkenazi Jewish Individuals. J. Genet. Couns..

[28] Bernstein-Molho R., Singer A., Laitman Y., Netzer I., Zalmanoviz S., Friedman E. (2019). Multigene panel testing in unselected Israeli breast cancer cases: Mutational spectrum and use of BRCA1/2 mutation prediction algorithms. Breast Cancer Res. Treat..

[29] Bąk A., Janiszewska H., Junkiert-Czarnecka A., Heise M., Pilarska-Deltow M., Laskowski R., Pasińska M., Haus O. (2014). A risk of breast cancer in women-carriers of constitutional CHEK2 gene mutations, originating from the North-Central Poland. Hered. Cancer Clin. Pract..

[30] Baralle D., Baralle M. (2005). Splicing in action: Assessing disease causing sequence changes. J. Med. Genet..

[31] Chen X., Truong T.T., Weaver J., Bove B.A., Cattie K., Armstrong B.A., Daly M.B., Godwin A.K. (2006). Intronic alterations in BRCA1 and BRCA2: Effect on mRNA splicing fidelity and expression. Hum. Mutat..

[32] Tedaldi G., Tebaldi M., Zampiga V., Danesi R., Arcangeli V., Ravegnani M., Cangini I., Pirini F., Petracci E., Rocca A. (2017). Multiple-gene panel analysis in a case series of 255 women with hereditary breast and ovarian cancer. Oncotarget.

[33] Lang G.T., Shi J.X., Hu X., Zhang C.H., Shan L., Song C.G., Zhuang Z.G., Cao A.Y., Ling H., Yu K.D. (2017). The spectrum of BRCA mutations and characteristics of BRCA-associated breast cancers in China: Screening of 2,991 patients and 1,043 controls by next-generation sequencing. Int. J. Cancer.

[34] Machackova E., Foretova L., Lukesova M., Vasickova P., Navratilova M., Coene I., Pavlu H., Kosinova V., Kuklova J., Claes K. (2008). Spectrum and characterisation of BRCA1 and BRCA2 deleterious mutations in high-risk Czech patients with breast and/or ovarian cancer. BMC Cancer.

[35] Coppa A., Buffone A., Capalbo C., Nicolussi A., D’Inzeo S., Belardinilli F., Colicchia V., Petroni M., Granato T., Midulla C. (2014). Novel and recurrent BRCA2 mutations in Italian breast/ovarian cancer families widen the ovarian cancer cluster region boundaries to exons 13 and 14. Breast Cancer Res. Treat..

[36] Parsons M.T., Tudini E., Li H., Hahnen E., Wappenschmidt B., Feliubadaló L., Aalfs C.M., Agata S., Aittomäki K., Alducci E. (2019). Large scale multifactorial likelihood quantitative analysis of BRCA1 and BRCA2 variants: An ENIGMA resource to support clinical variant classification. Hum. Mutat..

[37] Susswein L.R., Marshall M.L., Nusbaum R., Vogel Postula K.J., Weissman S.M., Yackowski L., Vaccari E.M., Bissonnette J., Booker J.K., Cremona M.L. (2016). Pathogenic and likely pathogenic variant prevalence among the first 10,000 patients referred for next-generation cancer panel testing. Genet. Med. Off. J. Am. Coll. Med. Genet..

[38] Borg A., Haile R.W., Malone K.E., Capanu M., Diep A., Törngren T., Teraoka S., Begg C.B., Thomas D.C., Concannon P. (2010). Characterization of BRCA1 and BRCA2 deleterious mutations and variants of unknown clinical significance in unilateral and bilateral breast cancer: The WECARE study. Hum. Mutat..

[39] De Brakeleer S., Bogdani M., De Grève J., Decock J., Sermijn E., Bonduelle M., Goelen G., Teugels E. (2007). Loss of nuclear BRCA1 protein staining in normal tissue cells derived from BRCA1 and BRCA2 mutation carriers. Mutat. Res..

[40] Kaneyasu T., Mori S., Yamauchi H., Ohsumi S., Ohno S., Aoki D., Baba S., Kawano J., Miki Y., Matsumoto N. (2020). Prevalence of disease-causing genes in Japanese patients with BRCA1/2-wildtype hereditary breast and ovarian cancer syndrome. NPJ Breast Cancer.

[41] Decker B., Allen J., Luccarini C., Pooley K.A., Shah M., Bolla M.K., Wang Q., Ahmed S., Baynes C., Conroy D.M. (2017). Rare, protein-truncating variants in ATM, CHEK2 and PALB2, but not XRCC2, are associated with increased breast cancer risks. J. Med. Genet..

[42] Yadav S., Reeves A., Campian S., Paine A., Zakalik D. (2017). Outcomes of retesting BRCA negative patients using multigene panels. Fam. Cancer.

[43] Liu X., Li H., Shao B., Wu J., Kong W., Song G., Jiang H., Wang J., Wan F. (2017). Identification of recurrent BRCA1 mutation and its clinical relevance in Chinese Triple-negative breast cancer cohort. Cancer Med..

[44] Giacomelli A.O., Yang X., Lintner R.E., McFarland J.M., Duby M., Kim J., Howard T.P., Takeda D.Y., Ly S.H., Kim E. (2018). Mutational processes shape the landscape of TP53 mutations in human cancer. Nat. Genet..

[45] Kotler E., Shani O., Goldfeld G., Lotan-Pompan M., Tarcic O., Gershoni A., Hopf T.A., Marks D.S., Oren M., Segal E. (2018). A Systematic p53 Mutation Library Links Differential Functional Impact to Cancer Mutation Pattern and Evolutionary Conservation. Mol. Cell.

[46] Lagerstedt-Robinson K., Rohlin A., Aravidis C., Melin B., Nordling M., Stenmark-Askmalm M., Lindblom A., Nilbert M. (2016). Mismatch repair gene mutation spectrum in the Swedish Lynch syndrome population. Oncol. Rep..

[47] Wang Y., Wu J., Li W., Li J., Liu R., Yang B., Li C., Jiang T. (2021). Retrospective investigation of hereditary syndromes in patients with medulloblastoma in a single institution. Child’s Nerv. Syst. ChNS Off. J. Int. Soc. Pediatric Neurosurg..

[48] Li M., Zhang J., Ouyang T., Li J., Wang T., Fan Z., Fan T., Lin B., Xie Y. (2016). Incidence of BRCA1 somatic mutations and response to neoadjuvant chemotherapy in Chinese women with triple-negative breast cancer. Gene.

[49] HGVS. http://hgvs.org.

[50] Biswas K., Das R., Alter B.P., Kuznetsov S.G., Stauffer S., North S.L., Burkett S., Brody L.C., Meyer S., Byrd R.A. (2011). A comprehensive functional characterization of BRCA2 variants associated with Fanconi anemia using mouse ES cell-based assay. Blood.

[51] Tiao G., Improgo M.R., Kasar S., Poh W., Kamburov A., Landau D.A., Tausch E., Taylor-Weiner A., Cibulskis C., Bahl S. (2017). Rare germline variants in ATM are associated with chronic lymphocytic leukemia. Leukemia.

[52] Yurgelun M.B., Kulke M.H., Fuchs C.S., Allen B.A., Uno H., Hornick J.L., Ukaegbu C.I., Brais L.K., McNamara P.G., Mayer R.J. (2017). Cancer Susceptibility Gene Mutations in Individuals With Colorectal Cancer. J. Clin. Oncol. Off. J. Am. Soc. Clin. Oncol..

[53] Bandeira G., Rocha K., Lazar M., Ezquina S., Yamamoto G., Varela M., Takahashi V., Aguena M., Gollop T., Zatz M. (2021). Germline variants of Brazilian women with breast cancer and detection of a novel pathogenic ATM deletion in early-onset breast cancer. Breast Cancer.

[54] Akcay I.M., Celik E., Agaoglu N.B., Alkurt G., Kizilboga Akgun T., Yildiz J., Enc F., Kir G., Canbek S., Kilic A. (2021). Germline pathogenic variant spectrum in 25 cancer susceptibility genes in Turkish breast and colorectal cancer patients and elderly controls. Int. J. Cancer.

[55] Kelsen J.R., Dawany N., Moran C.J., Petersen B.S., Sarmady M., Sasson A., Pauly-Hubbard H., Martinez A., Maurer K., Soong J. (2015). Exome sequencing analysis reveals variants in primary immunodeficiency genes in patients with very early onset inflammatory bowel disease. Gastroenterology.

[56] Hauke J., Horvath J., Groß E., Gehrig A., Honisch E., Hackmann K., Schmidt G., Arnold N., Faust U., Sutter C. (2018). Gene panel testing of 5589 BRCA1/2-negative index patients with breast cancer in a routine diagnostic setting: Results of the German Consortium for Hereditary Breast and Ovarian Cancer. Cancer Med..

[57] Shindo K., Yu J., Suenaga M., Fesharakizadeh S., Cho C., Macgregor-Das A., Siddiqui A., Witmer P.D., Tamura K., Song T.J. (2017). Deleterious Germline Mutations in Patients With Apparently Sporadic Pancreatic Adenocarcinoma. J. Clin. Oncol. Off. J. Am. Soc. Clin. Oncol..

[58] Kraus C., Hoyer J., Vasileiou G., Wunderle M., Lux M.P., Fasching P.A., Krumbiegel M., Uebe S., Reuter M., Beckmann M.W. (2017). Gene panel sequencing in familial breast/ovarian cancer patients identifies multiple novel mutations also in genes others than BRCA1/2. Int. J. Cancer.

[59] Akbari M.R., Malekzadeh R., Lepage P., Roquis D., Sadjadi A.R., Aghcheli K., Yazdanbod A., Shakeri R., Bashiri J., Sotoudeh M. (2011). Mutations in Fanconi anemia genes and the risk of esophageal cancer. Hum. Genet..

[60] Delimitsou A., Fostira F., Kalfakakou D., Apostolou P., Konstantopoulou I., Kroupis C., Papavassiliou A.G., Kleibl Z., Stratikos E., Voutsinas G.E. (2019). Functional characterization of CHEK2 variants in a Saccharomyces cerevisiae system. Hum. Mutat..

[61] Ciceri S., Gamba B., Corbetta P., Mondini P., Terenziani M., Catania S., Nantron M., Bianchi M., D’Angelo P., Torri F. (2018). Genetic and epigenetic analyses guided by high resolution whole-genome SNP array reveals a possible role of CHEK2 in Wilms tumour susceptibility. Oncotarget.

[62] Isaacsson Velho P., Silberstein J.L., Markowski M.C., Luo J., Lotan T.L., Isaacs W.B., Antonarakis E.S. (2018). Intraductal/ductal histology and lymphovascular invasion are associated with germline DNA-repair gene mutations in prostate cancer. Prostate.

[63] Young E.L., Feng B.J., Stark A.W., Damiola F., Durand G., Forey N., Francy T.C., Gammon A., Kohlmann W.K., Kaphingst K.A. (2016). Multigene testing of moderate-risk genes: Be mindful of the missense. J. Med. Genet..

[64] Le Calvez-Kelm F., Lesueur F., Damiola F., Vallée M., Voegele C., Babikyan D., Durand G., Forey N., McKay-Chopin S., Robinot N. (2011). Rare, evolutionarily unlikely missense substitutions in CHEK2 contribute to breast cancer susceptibility: Results from a breast cancer family registry case-control mutation-screening study. Breast Cancer Res. BCR.

[65] Liccardo R., De Rosa M., Rossi G.B., Carlomagno N., Izzo P., Duraturo F. (2017). Incomplete Segregation of MSH6 Frameshift Variants with Phenotype of Lynch Syndrome. Int. J. Mol. Sci..

[66] Baloch A.H., Daud S., Raheem N., Luqman M., Ahmad A., Rehman A., Shuja J., Rasheed S., Ali A., Kakar N. (2014). Missense mutations (p.H371Y, p.D438Y) in gene CHEK2 are associated with breast cancer risk in women of Balochistan origin. Mol. Biol. Rep..

[67] Tischkowitz M.D., Yilmaz A., Chen L.Q., Karyadi D.M., Novak D., Kirchhoff T., Hamel N., Tavtigian S.V., Kolb S., Bismar T.A. (2008). Identification and characterization of novel SNPs in CHEK2 in Ashkenazi Jewish men with prostate cancer. Cancer Lett..

[68] Moradian M.M., Babikyan D.T., Markarian S., Petrosyan J.G., Avanesian N., Arutunyan T., Sarkisian T.F. (2021). Germline mutational spectrum in Armenian breast cancer patients suspected of hereditary breast and ovarian cancer. Hum. Genome Var..

[69] Dominguez-Valentin M., Nakken S., Tubeuf H., Vodak D., Ekstrøm P.O., Nissen A.M., Morak M., Holinski-Feder E., Holth A., Capella G. (2019). Results of multigene panel testing in familial cancer cases without genetic cause demonstrated by single gene testing. Sci. Rep..

[70] Scarpitta R., Zanna I., Aretini P., Gambino G., Scatena C., Mei B., Ghilli M., Rossetti E., Roncella M., Congregati C. (2019). Germline investigation in male breast cancer of DNA repair genes by next-generation sequencing. Breast Cancer Res. Treat..

[71] Ansari N., Shahrabi S., Khosravi A., Shirzad R., Rezaeean H. (2019). Prognostic Significance of CHEK2 Mutation in Progression of Breast Cancer. Lab. Med..

[72] Grandval P., Fabre A.J., Gaildrat P., Baert-Desurmont S., Buisine M.P., Ferrari A., Wang Q., Béroud C., Olschwang S. (2013). UMD-MLH1/MSH2/MSH6 databases: Description and analysis of genetic variations in French Lynch syndrome families. Database J. Biol. Databases Curation.

[73] Simbolo M., Mafficini A., Agostini M., Pedrazzani C., Bedin C., Urso E.D., Nitti D., Turri G., Scardoni M., Fassan M. (2015). Next-generation sequencing for genetic testing of familial colorectal cancer syndromes. Hered. Cancer Clin. Pract..

[74] Baudi F., Quaresima B., Grandinetti C., Cuda G., Faniello C., Tassone P., Barbieri V., Bisegna R., Ricevuto E., Conforti S. (2001). Evidence of a founder mutation of BRCA1 in a highly homogeneous population from southern Italy with breast/ovarian cancer. Hum. Mutat..

[75] Tung N., Lin N.U., Kidd J., Allen B.A., Singh N., Wenstrup R.J., Hartman A.R., Winer E.P., Garber J.E. (2016). Frequency of Germline Mutations in 25 Cancer Susceptibility Genes in a Sequential Series of Patients With Breast Cancer. J. Clin. Oncol. Off. J. Am. Soc. Clin. Oncol..

[76] Lee C.H., Chung J.H. (2001). The hCds1 (Chk2)-FHA domain is essential for a chain of phosphorylation events on hCds1 that is induced by ionizing radiation. J. Biol. Chem..

[77] Schwarz J.K., Lovly C.M., Piwnica-Worms H. (2003). Regulation of the Chk2 protein kinase by oligomerization-mediated cis- and trans-phosphorylation. Mol. Cancer Res. MCR.

[78] Yehia L., Ni Y., Sesock K., Niazi F., Fletcher B., Chen H.J.L., LaFramboise T., Eng C. (2018). Unexpected cancer-predisposition gene variants in Cowden syndrome and Bannayan-Riley-Ruvalcaba syndrome patients without underlying germline PTEN mutations. PLoS Genet..

[79] Bono M., Fanale D., Incorvaia L., Cancelliere D., Fiorino A., Calò V., Dimino A., Filorizzo C., Corsini L.R., Brando C. (2021). Impact of deleterious variants in other genes beyond BRCA1/2 detected in breast/ovarian and pancreatic cancer patients by NGS-based multi-gene panel testing: Looking over the hedge. ESMO Open.

[80] Pylkäs K., Tommiska J., Syrjäkoski K., Kere J., Gatei M., Waddell N., Allinen M., Karppinen S.M., Rapakko K., Kääriäinen H. (2007). Evaluation of the role of Finnish ataxia-telangiectasia mutations in hereditary predisposition to breast cancer. Carcinogenesis.

[81] Tram E., Savas S., Ozcelik H. (2013). Missense variants of uncertain significance (VUS) altering the phosphorylation patterns of BRCA1 and BRCA2. PLoS ONE.

[82] Couch F.J., DeShano M.L., Blackwood M.A., Calzone K., Stopfer J., Campeau L., Ganguly A., Rebbeck T., Weber B.L. (1997). BRCA1 mutations in women attending clinics that evaluate the risk of breast cancer. New Engl. J. Med..

[83] Sermijn E., Goelen G., Teugels E., Kaufman L., Bonduelle M., Neyns B., Poppe B., De Paepe A., De Grève J. (2004). The impact of proband mediated information dissemination in families with a BRCA1/2 gene mutation. J. Med. Genet..

[84] Figlioli G., De Nicolo A., Catucci I., Manoukian S., Peissel B., Azzollini J., Beltrami B., Bonanni B., Calvello M., Bondavalli D. (2021). Analysis of Italian BRCA1/2 pathogenic variants identifies a private spectrum in the population from the Bergamo province in Northern Italy. Cancers.

[85] Li N., Zethoven M., McInerny S., Healey E., DeSilva D., Devereux L., Scott R.J., James P.A., Campbell I.G. (2022). Contribution of large genomic rearrangements in PALB2 to familial breast cancer: Implications for genetic testing. J. Med. Genet..

[86] Li J.Y., Jing R., Wei H., Wang M., Xiaowei Q., Liu H., Jian L., Ou J.H., Jiang W.H., Tian F.G. (2019). Germline mutations in 40 cancer susceptibility genes among C hinese patients with high hereditary risk breast cancer. Int. J. Cancer.

[87] Tommiska J., Jansen L., Kilpivaara O., Edvardsen H., Kristensen V., Tamminen A., Aittomäki K., Blomqvist C., Børresen-Dale A.L., Nevanlinna H. (2006). ATM variants and cancer risk in breast cancer patients from Southern Finland. BMC Cancer.

[88] Goldgar D.E., Healey S., Dowty J.G., Da Silva L., Chen X., Spurdle A.B., Terry M.B., Daly M.J., Buys S.M., Southey M.C. (2011). Rare variants in the ATM gene and risk of breast cancer. Breast Cancer Res. BCR.

[89] Kleiblova P., Stolarova L., Krizova K., Lhota F., Hojny J., Zemankova P., Havranek O., Vocka M., Cerna M., Lhotova K. (2019). Identification of deleterious germline CHEK2 mutations and their association with breast and ovarian cancer. Int. J. Cancer.

[90] Terkelsen T., Christensen L.-L., Fenton D.C., Jensen U.B., Sunde L., Thomassen M., Skytte A.-B. (2019). Population frequencies of pathogenic alleles of BRCA1 and BRCA2: Analysis of 173 Danish breast cancer pedigrees using the BOADICEA model. Fam. Cancer.

[91] Antoniou A.C., Hardy R., Walker L., Evans D.G., Shenton A., Eeles R., Shanley S., Pichert G., Izatt L., Rose S. (2008). Predicting the likelihood of carrying a BRCA1 or BRCA2 mutation: Validation of BOADICEA, BRCAPRO, IBIS, Myriad and the Manchester scoring system using data from UK genetics clinics. J. Med. Genet..

[92] O’leary E., Iacoboni D., Holle J., Michalski S.T., Esplin E.D., Yang S., Ouyang K. (2017). Expanded gene panel use for women with breast cancer: Identification and intervention beyond breast cancer risk. Ann. Surg. Oncol..

