# Supplementary material for: Germline Testing in a Cohort of Patients at High Risk of Hereditary Cancer Predisposition Syndromes: First Two-Year Results from South Italy
Source: Genes (Basel). 2022 Jul 21;13(7):1286. doi: 10.3390/genes13071286 (PMC9319682; doi:10.3390/genes13071286)
Supplement: Supplementary file 1 [file genes-13-01286-s001.zip › Supplementary Figure S1.pdf]

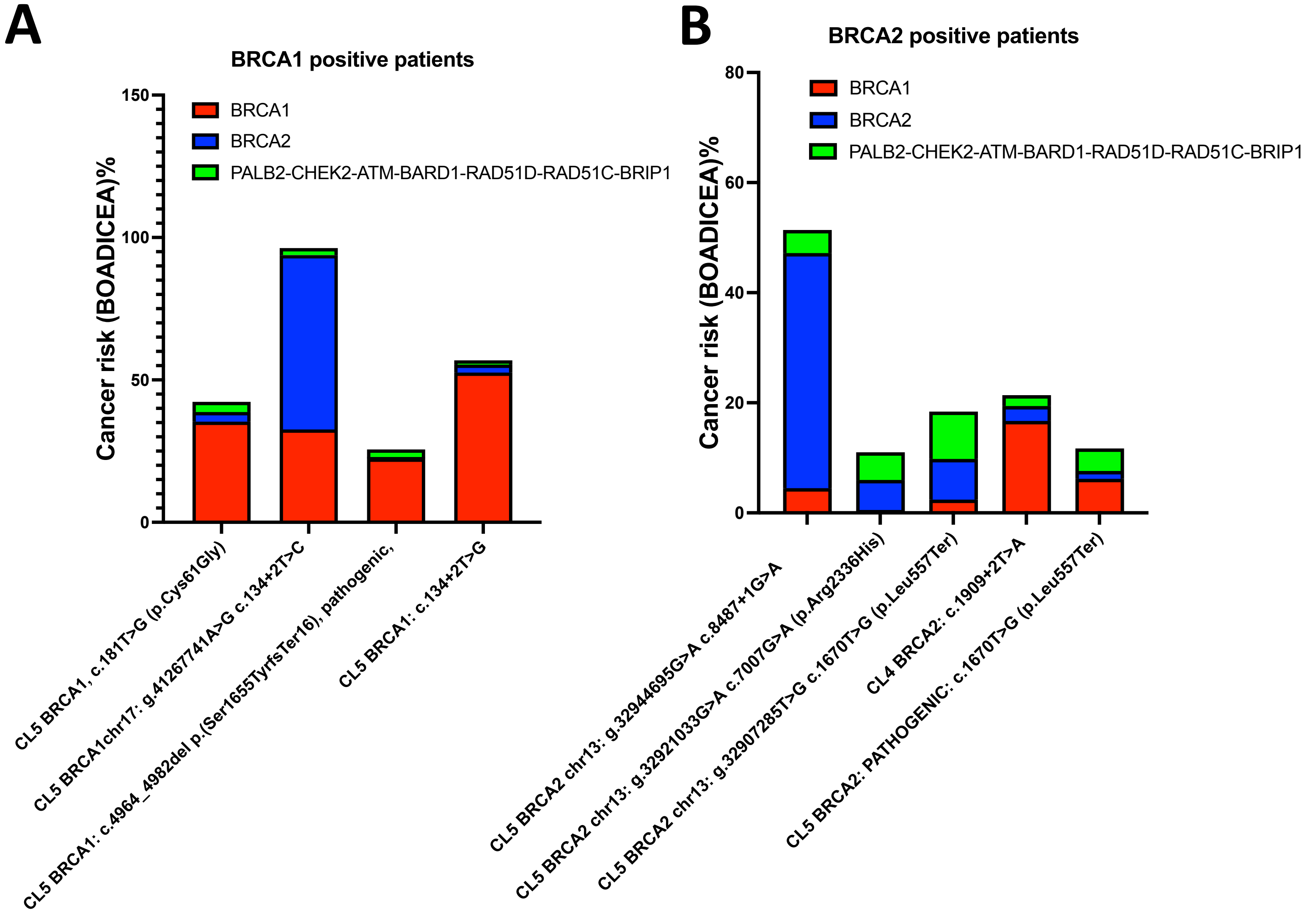

Supplementary Figure S1: A) Likelihood of carrying PVs in *BRCA1* positive patients evaluated by BOADICEA for *BRCA1* and *BRCA2* and other susceptibility genes. **B)** Likelihood of carrying pathogenic variants in *BRCA2* positive patients evaluated by the BOADICEA for *BRCA1* and *BRCA2* and other susceptibility genes.
