# Supplementary material for: Germline Testing in a Cohort of Patients at High Risk of Hereditary Cancer Predisposition Syndromes: First Two-Year Results from South Italy
Source: Genes (Basel). 2022 Jul 21;13(7):1286. doi: 10.3390/genes13071286 (PMC9319682; doi:10.3390/genes13071286)
Supplement: Supplementary file 1 [file genes-13-01286-s001.zip › Supplementary Table S2.pdf]

Supplementary Table S2. Interpretation of VUS variants using ACMG rules and semiquantitative rules by Sherloc.

n.	Patient ID	Gene	Variant	ACMG interpretation	Sherloc interpretation
**1**	**674/19**	*ATM*	c.7475T>G (p.Leu2492Arg)	PM2, PP3, BP6: *VUS*	1B, 2P: *uncertain significance*
**2**	**348/20**	*ATM*	c.3356C>T (p.Ala1119Val)	PM2: *VUS*	2,5P: *uncertain significance*
**3**	**704/20**	*ATM*	c.7316T>C (p.Val2439Ala)	PM2: *VUS*	1,5P,1B: *uncertain significance*
**4**	**87/21**	*PALB2*	c.37G>A (p.Glu13Lys)	PM2: *VUS*	3P: *uncertain significance*
**5**	**133/21**	*CHEK2*	c.911T>C (p.Met304Thr)	PM2, PP3: *VUS*	3P: *uncertain significance*
**6**	**150/21**	*BRCA1*	c.1344C>G (p.His448Gln)	PM2, BP4: *VUS*	2P,1B: *uncertain significance*
**7**	**182/21**	*BRCA1*	c.5312C>A (p.Pro1771His)	PM1, PM2, PP3: *VUS*	3P: *uncertain significance*
**8**	**262/21**	*MSH6*	c.2941A>G (p.Ile981Val)	PM2, BP4: *VUS*	2P,1B: *uncertain significance*
**9**	**282/21**	*CHEK2*<br>*MLH1*<br>*MSH6*	1312G>T (p.Asp438Tyr)<br>c.678-4A>G<br>c.998C>T (p.Thr333Ile)	PS3, PM2, BS3: *VUS*<br>PM2, BP4: *VUS*<br>PM2: *VUS*	2P,1B: *uncertain significance*<br>1P,1B: *uncertain significance*<br>2P: *uncertain significance*
**10**	**310/21**	*PALB2*	c.563C>G (p.Ala188Gly)	PM2, BP4: *VUS*	2P,1B: *uncertain significance*
**11**	**344/21**	*CHEK2*	c.1312G>T (p.Asp438Tyr)	PS3, PM2, BS3: *VUS*	2P,1B: *uncertain significance*
**12**	**465/21**	*ATM*	c.2954A>G (p.Asp985Gly)	PM2: *VUS*	2,5P: *uncertain significance*
**13**	**489/21**	*MSH6*	c.1311_1312delinsGC(p. 437_438delinsGlnLeu)	PM2, PP3: VUS	2P: uncertain significance
**14**	**620/21**	*CHEK2*	c.787G>C (p.Glu263Gln)	PM2: *VUS*	2P: *uncertain significance*
**15**	**665/21**	*CHEK2*	c.1160C>T (p.Thr387Ile)	PM2, PP3: *VUS*	2,5P: *uncertain significance*
**16**	**760/21**	*PMS2*	c.328G>T(p.Ala110Ser)	PM2, PP3: *VUS*	2,5P: *uncertain significance*
**17**	**761/21**	*PMS2*	c.328G>T(p.Ala110Ser)	PM2, PP3: *VUS*	2,5P: *uncertain significance*
**18**	**979/21**	*CHEK2*<br>*MLH1*	c.-4C>T<br>c.2130C>G (p.Asn710Lys)	BP4, BP7, PM2: LB<br>PM2, BP4: *VUS*	3B: *uncertain significance*<br>1,5P,1B*: uncertain significance*
**19**	**1006/21**	*ATM*	c.7316T>C (p.Val2439Ala)	PM2: VUS	1,5P: uncertain significance
**20**	**68/22**	*ATM*	c.8734A>G (p.Arg2912Gly)	PP5, PM2, PP3: VUS	3P,1B: uncertain significance
**21**	**156/22**	BRCA1	c.1250A>G (p.Asn417Ser)	PM2, BP4: VUS	2P,1B: uncertain significance

Detailed sherlock criteria used for the classification are described in reference *[46]*.
